# SENP3 in monocytes/macrophages up‐regulates tissue factor and mediates lipopolysaccharide‐induced acute lung injury by enhancing JNK phosphorylation

**DOI:** 10.1111/jcmm.15199

**Published:** 2020-03-31

**Authors:** Xuelian Chen, Yimin Lao, Jing Yi, Jie Yang, Shuangjun He, Yi Chen

**Affiliations:** ^1^ Emergency Department South Campus, Renji Hospital Shanghai Jiaotong University School of Medicine Shanghai China; ^2^ Shanghai Key Laboratory of Tumor Microenvironment and Inflammation Department of Biochemistry and Molecular Cell Biology Institutes of Medical Sciences Shanghai Jiao Tong University School of Medicine Shanghai China

**Keywords:** coagulation, macrophages, monocytes, reactive oxygen species, SENP3, tissue factor

## Abstract

The mechanisms underlying coagulation abnormalities in sepsis and septic acute lung injury remain unclear. Tissue factor (TF) initiates coagulation; its production can be regulated by reactive oxygen species (ROS); and monocytes/macrophages produce pathological TF during sepsis. The SUMO2/3 protease SENP3 is redox‐sensitive, and SENP3 accumulation in lipopolysaccharide (LPS)‐activated macrophages is ROS‐dependent. To explore whether SENP3 contributes to LPS‐activated coagulation, we used mice with *Senp3* conditional knockout (cKO) in myeloid cells. In the model of LPS‐induced sepsis, SENP3 cKO mice exhibited less severe acute lung injury than SENP3 ^fl/fl^ mice. SENP3 cKO mice exhibited decreased TF expression in monocytes and alveolar macrophages, with consequently compromised coagulation in their blood and lungs. In vitro results showed that ROS‐induced SENP3 accumulation contributed to LPS‐induced TF expression, which was reduced by JNK inhibitor SP600125. Furthermore, mice injected with LPS following SP600125 (75 mg/kg) treatment showed decreased monocytes/macrophages TF production and alleviated coagulation activation, with less severe lung injury and higher survival rates. Collectively, the results suggest that SENP3 mediates LPS‐induced coagulation activation by up‐regulating monocyte/macrophage TF production in a JNK‐dependent manner. This work provides new insights into ROS regulation of LPS‐activated coagulation and reveals a link between SUMOylation and coagulation.

## 1 INTRODUCTION

Coagulation abnormalities contribute to the lethality of sepsis and septic acute lung injury (ALI).^1^ To date, all methods aimed at alleviating coagulation activation during sepsis failed to improve mortality rates in clinical trials, mainly due to compromised haemostasis and bleeding complications.^2^, ^3^ Tissue factor (TF) is a primary trigger for coagulation and thrombus formation.^4^ Aberrant expression of TF plays a pivotal role in sepsis‐associated blood clotting activation.^7^ As recent studies indicate that monocyte/macrophage TF rather than endothelial TF induces pathological coagulation in sepsis,^5^, ^6^ strategies that selectively inhibit TF expression in monocytes/macrophages, without affecting TF activity in extravascular cells, could decrease pathological activation of coagulation and minimize bleeding complications in sepsis.^7^ Therefore, studying the mechanism of TF production in monocytes/macrophages during sepsis is crucial.

Monocytes/macrophages produce high levels of reactive oxygen species (ROS) during the microbial invasion, which are required for both pathogen clearance and intracellular signalling.^8^ During Gram‐negative sepsis, lipopolysaccharide (LPS) activates toll‐like receptor (TLR) 4, which acts as the second messenger to activate ROS‐sensitive signalling pathways like mitogen‐activated protein kinase (MAPK), including extracellular signal‐regulated protein kinases (ERK), p38 MAP kinase and c‐Jun NH2‐terminal kinases (JNK1/2).^9^ Although many studies have analysed ROS‐regulated TF expression in macrophages,^10^ the underlying mechanism is largely unknown.

SUMOylation, an important post‐translational protein modification, can regulate various aspects of protein biology, such as stability, activity or localization, thereby modulating gene expression and cellular functionality in a specific manner.^11^ SUMOylation is a dynamic process that can be reversed by SUMO‐specific proteases (SENP) to maintain the balance of SUMOylated and deSUMOylated proteins.^12^ The SUMO protease SENP3, which removes SUMO2/3 from target proteins, is sensitive to oxidative stress.^13^, ^14^ ROS can lead to rapid increases in SENP3 nuclear levels by inhibiting its proteasomal degradation.^13^, ^14^ Lao's study proves that SENP3 potentiates LPS‐induced TLR4 signalling via deSUMOylation of mitogen‐activated protein kinase kinase 7 (MKK7) leading to enhanced JNK phosphorylation and related downstream events.^15^

Thus, we presumed that SENP3 might contribute to LPS‐induced monocytes/macrophages coagulation activation via ROS‐sensitive JNK pathway. To test this hypothesis, we used SENP3 flox/flox^(fl/fl)^ Lyz2‐cre mice (mice with *Senp3* conditional knockout [cKO] in myeloid cells, hereafter named SENP3 cKO mice) to investigate the role of SENP3 in regulating LPS‐induced TF expression and coagulation status in vivo. The results showed that SENP3 deficiency contributed to less severe LPS‐induced ALI and alleviated coagulation. In vitro results showed that SENP3 deficiency reduced TF expression. JNK inhibition was sufficient to reduce TF expression in response to LPS stimulation in vitro and in vivo. Therefore, this study verifies that SENP3 plays a role in mediating LPS‐induced TF expression in a JNK‐dependent manner in monocytes/macrophages.

## 2 MATERIALS AND METHODS

### 2.1 Animal care

Six‐ to eight‐week‐old C57BL/6 wild‐type (WT) mice from the Shanghai SLAC Laboratory Animal Co. Ltd. were housed under specific pathogen‐free conditions with free access to food and water. C57BL/6 SENP3^fl/fl^ and Lyz2‐Cre SENP3 cKO mice were generated as previously detailed.^15^ The Guide for the Care and Use of Laboratory Animals issued by the Ministry of Science and Technology of the People's Republic of China was followed for all animal studies, which received approval from the Institutional Animal Care & Use Committee of Shanghai Jiao Tong University School of Medicine (Permit Number: A‐2016‐023). Sodium pentobarbital was used to anaesthetize animals during surgery, and all possible efforts were made to reduce suffering.

### 2.2 LPS‐induced sepsis model

SENP3^fl/fl^ and SENP3 cKO mice were intraperitoneally (i.p.) injected with 30 mg/kg LPS (Sigma‐Aldrich). The blood and plasma were collected at 2, 4 and 8 hours post‐LPS injection. After 8 hours, the left lungs were isolated for histopathologic evaluation, whereas bronchoalveolar lavage fluid (BALF) was collected from the right lungs. JNK inhibitor SP600125 (APEXBIO) was dissolved in dimethyl sulfoxide (DMSO) at 15 mg/mL. Male C57BL/6 mice were i.p. injected with 30mg/kg LPS. After 15 minutes, these mice were i.p. injected with SP600125 (75 mg/kg) or DMSO. Mouse survival from LPS injection following SP600125 treatment or DMSO was monitored every 4 hours for up to 60 hours after LPS injection. The blood and BALF were obtained at 4 hours post‐LPS injection. After 8 hours, the total lungs were isolated for histopathologic evaluation.

### 2.3 Histopathologic evaluation

The haematoxylin and eosin (H&E) staining was carried out following the regular staining method, using the left lungs or total lungs.^15^ The histopathologic evaluation was finished by two independent pathologists and accessed using a semi‐quantitative scoring method.^16^

### 2.4 Lung wet‐to‐dry weight ratio and total protein concentration in the BALF

The right lungs were removed, each lung was blotted dry, weighed and then placed in an oven at 70°C for 48 hours to obtain the ‘dry’ weight. The total protein concentration in the BALF was measured using BCA Protein Assay Kit.^17^

### 2.5 Cell culture and transfection

RAW264.7 cells were cultured in Dulbecco's modified Eagle's medium (HyClone) with 10% foetal bovine serum (FBS; Gibco), 100 units/mL of penicillin and 100 mg/L of streptomycin (Gibco), at 37°C in a humidified atmosphere with 5% CO_2_. SENP3‐specific (si‐SENP3 [sense]: 5′‐GGGCUGGAAAGGUUACUUCAAdTdT‐3′) and non‐specific siRNA oligoes were transfected into RAW264.7 cells with Attractene Transfection Reagent (Qiagen) following the manufacturer's instructions.SP600125 (Abcam) was used as the JNK kinase inhibitor in cell experiments.

### 2.6 Bone marrow‐derived macrophage culture

Bone marrow‐derived macrophages (BMDMs) were isolated from SENP3^fl/fl^ Lyz2‐Cre mouse bone marrow. Freshly isolated bone marrow cells were plated in RPMI 1640 containing 2 mmol/L l‐glutamine, 10% FBS, penicillin/streptomycin and 50 ng/mL murine macrophage colony‐stimulating factor (M‐CSF; Peprotech). Cells were collected for use on day 7. WT murine BMDMs were pre‐treated with antioxidant N‐acetyl‐L‐cysteine (NAC; Sigma‐Aldrich) for 4 hours before LPS stimulation in some experiments.

### 2.7 Quantitative RT‐PCR

The total RNA was isolated following the TRIzol reagent kit protocol (Invitrogen). The cDNA synthesis was performed on 0.5 μg of the total RNA with a reverse transcription kit (Takara). Real‐time PCR was conducted on the ABI Prism 7500 system using SYBR Green (Takara) and following the manufacturer's instructions, with GAPDH as a normalization control. The following primers were used: mouse TF forward: 5′‐CATCCTCCTGTCCATATCTCTGTG‐3′; mouse TF reverse: 5′‐GTGACCTAGTGTCTTCCTGAGTG‐3′; mice GAPDH forward: 5′‐TGTGTCCGTCGTGGATCTGA‐3′; mice GAPDH reverse: 5′‐CCTGCTTCACCACCTTCTTG‐3′.

### 2.8 Immunoblotting

Immunoblotting (IB) was conducted as in previous studies.^15^ Antibodies specific for SENP3 (5591), JNK (9252), phospho‐SPAK/JNK (Thr183/Tyr185) (4668), p38 (8690), phospho‐p38 (Thr180/Tyr182) (4511), p44/42 MAPK (Erk1/2) (4695) and phospho‐ERK (Thr202/Tyr204) (4370) were all purchased from Cell Signaling Technology. Anti‐murine TF antibody was purchased from R&D Systems (AF3178).

### 2.9 ELISA

Bronchoalveolar lavage fluid IL‐6, TNF‐α and IL‐1β levels were assessed using murine ELISA kits based on provided directions. TAT complex levels were assessed with Thrombin‐Antithrombin Complex ELISA kits (Abcam). For analysis of plasma TAT levels, blood was collected from the inferior vena cava into sodium citrate (final concentration 0.38%) and spun at 2000 g for 10 minutes before collecting plasma, which was stored at −80°C. For BALF collection, 0.6 mL of PBS was slowly instilled into the lung and then removed, with the resultant BALF stored at −80℃ in aliquots.

### 2.10 Immunofluorescence

Lungs were isolated, and frozen sections were sliced (10 μm). Samples were permeabilized using 0.2% Triton X‐100 before blocking using 20% goat serum and then probed with primary antibodies at 4°C overnight. For TF staining, the same primary antibodies were used as in IB experiments, with Alexa Flour 555‐conjugated secondary antibody (Invitrogen). For F4/80 staining, the antibody was obtained from eBioscience, with Alexa Flour 488‐conjugated secondary antibody (Invitrogen). DAPI was used for nuclear staining, and a LSM 710 fluorescent microscope (Zeiss) was used for imaging. The F4/80‐positive macrophages located in the lung interstitium were excluded. Five alveolar macrophages per mouse, which located in the alveolar region (n = 6; thus, 30 cells in total), were assessed.

### 2.11 Flow cytometry

Blood was collected from venous plexus inside the orbit behind the eyeball and subjected to Red Blood Cell Lysing Buffer for 5 minutes. Cells obtained from blood, and BALF samples were stained with appropriate primary antibodies on ice in the dark for 30 minutes, followed by washing and analysis on an LSRII flow cytometer (BD Biosciences). FACSDiva (version 6.1.1; BD Biosciences) and Prism 5.0 (GraphPad) were used for data analysis. Antibodies specific for CD11b (566 416) and F4/80 (743 280) were purchased from BD Biosciences. Mouse TF PE‐conjugated antibody (FAB3178P) was purchased from R&D systems.

### 2.12 Statistical analysis

Student's *t* test was used to determine the significance of the differences between two or more groups of data. Survival curves were generated using the Kaplan‐Meier method, and the significance of difference was calculated by the log‐rank test. A value of *P* < .05 was considered statistically significant.

## 3 RESULTS

### 3.1 SENP3 cKO mice exhibited less severe LPS‐induced acute lung injury

**FIGURE 1:**
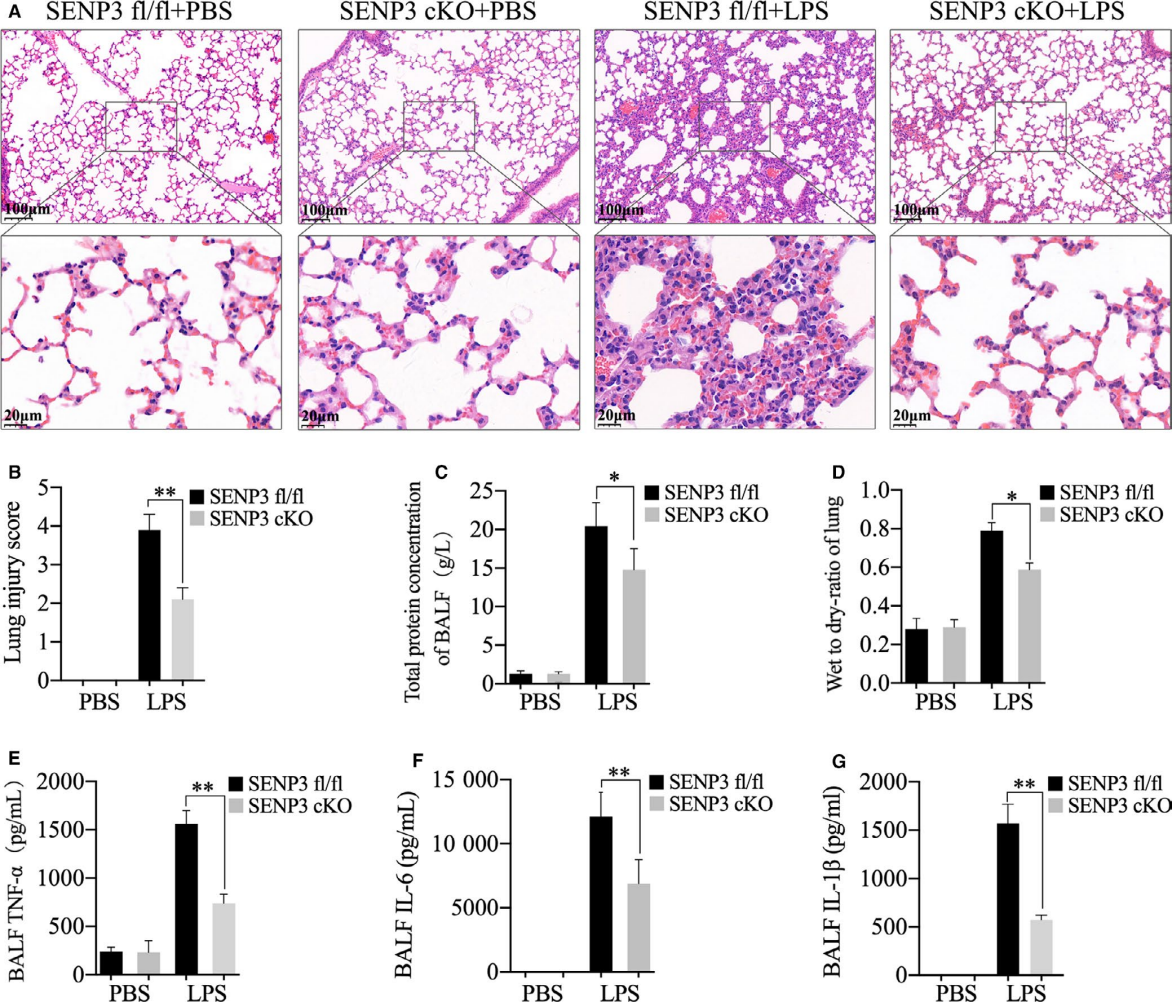
SENP3 conditional knockout (cKO) mice exhibited less severe lipopolysaccharide (LPS)‐induced acute lung injury. A‐G, SENP3^fl/fl^ and cKO mice (n = 6) were injected i.p. using LPS (30 mg/kg) for 8 h. A, H&E staining, (B) total lung injury score, (C) total bronchoalveolar lavage fluid (BALF) protein levels and (D) wet‐to‐dry ratios were assessed. E, BALF IL‐6, (F) TNF‐α and (G) IL‐1β levels were assessed by ELISA. Graphs show the mean ± SD, and data (B‐G) shown are representative of three independent experiments. *P < .05; **P < .01

Our previous study showed that SENP3 cKO mice had much higher survival rates than the SENP3^fl/fl^ mice.^15^To assess how a lack of myeloid SENP3 expression affected the severity of septic ALI, we used the same mouse model of LPS‐induced sepsis. H&E staining of lung sections from SENP3^fl/fl^ mice revealed that LPS intraperitoneal injection induced ALI characterized by thickening of the alveolar septal membrane, alveolar oedema and inflammatory cell infiltration into the interstitial and alveolar spaces. By contrast, lung sections from SENP3 cKO mice exhibited fewer abnormalities, with some areas showing no detectable evidence of ALI (Figure 1A). The total lung injury score after LPS injection was significantly higher in SENP3^fl/fl^ than in SENP3 cKO mice (Figure 1B). Following LPS injection, total BALF protein levels and lung wet‐to‐dry weight (W/D) ratios increased both in SENP3^fl/fl^ mice and in SENP3 cKO mice, but the increase was significantly higher in SENP3^fl/fl^ than SENP3 cKO mice (Figure 1C,D). This suggests that SENP3 cKO animals suffered from less alveolar protein leakage. In addition, we assessed levels of three key pro‐inflammatory cytokines (IL‐6, TNF‐α and IL‐1β) by ELISA. Results showed that, following LPS injection, cytokine levels were significantly higher in the BALF of SENP3^fl/fl^ mice than SENP3 cKO mice (Figure 1E‐G). Collectively, these results revealed that SENP3 cKO mice suffered less severe LPS‐induced ALI than SENP3^fl/fl^ mice.

### 3.2 SENP3 cKO mice exhibited alleviated coagulation activation along with decreased TF production in monocytes and alveolar macrophages during LPS‐induced acute lung injury

**FIGURE 2:**
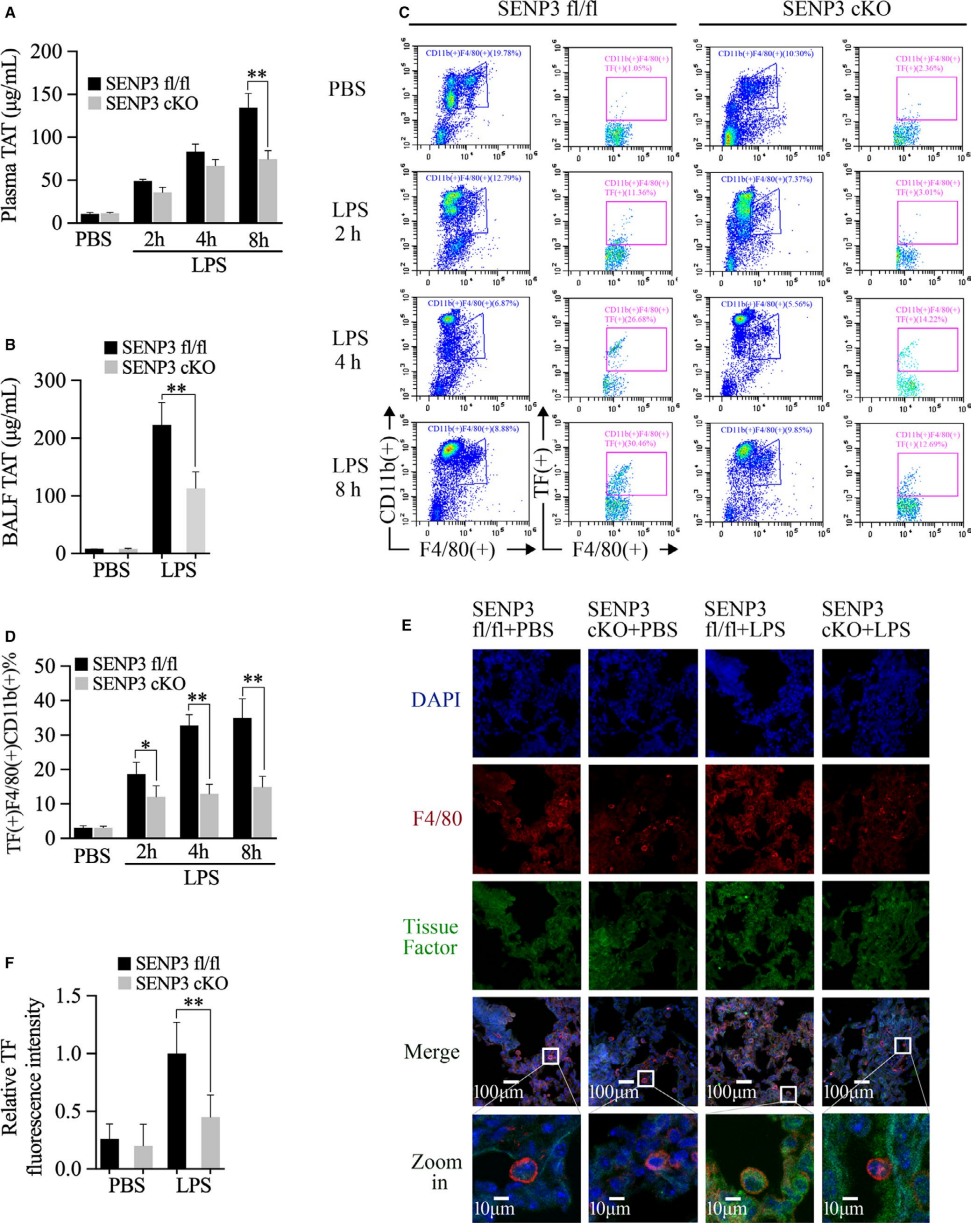
SENP3 conditional knockout (cKO) mice exhibited alleviated coagulation activation along with decreased tissue factor (TF) production in monocytes and alveolar macrophages during lipopolysaccharide (LPS)‐induced acute lung injury. SENP3^fl/fl^ and SENP3 cKO mice (n = 6) were i.p. injected using LPS (30 mg/kg). A, The levels of thrombin‐antithrombin (TAT) complexes in the plasma were assessed by ELISA. B, The levels of TAT complexes in the bronchoalveolar lavage fluid (BALF) were assessed by ELISA. C, TF in CD11b (+) F4/80 (+) peripheral blood cells was assessed by flow cytometry. D, The statistics of the frequency of TF (+) CD11b (+) F4/80 (+) cells. E, Staining for TF and F4/80 was visualized in lung tissue sections via confocal microscopy, with representative images shown and TF staining intensity quantified. TF: green; F4/80: red; DAPI: blue. Scale bar, 10 or 100 μm. F, Quantification of TF fluorescence intensity is shown. Five F4/80 (+) cells were analysed per mouse (n = 6). Graphs show the mean ± SD, and data (A‐D, F) shown are representative of three independent experiments. *P < .05; **P < .01

Extensive activation of the coagulation cascade is a central driver of sepsis‐associated organ dysfunction. Thus, we assessed coagulation induction via measuring thrombin‐antithrombin (TAT) complex levels in LPS‐injected mice.^6^ We found that LPS significantly increased plasma TAT complex levels in SENP3^fl/fl^ mice over time (Figure 2A). Moreover, plasma TAT complex levels at 8 hours after LPS injection were significantly higher in SENP3^fl/fl^ than in cKO mice. Accordingly, BALF TAT complex levels in SENP3 KO mice following LPS injection were significantly lower than in fl/fl controls (Figure 2B).

Previous studies have implicated that myeloid and haematopoietic cell TF was linked to TAT complex levels in the plasma and lung.^5^, ^6^, ^18^ Therefore, we sought to determine whether a lack of myeloid SENP3 altered TF expression on monocytes and alveolar macrophages in vivo. We first assessed TF expression on CD11b (+) F4/80 (+) cells in the peripheral blood via flow cytometry. After LPS injection, the frequency of CD11b (+) F4/80 (+) TF (+) cells increased in both SENP3 ^fl/fl^ and cKO mice. Importantly, at 2, 4 and 8 hours, the proportion of CD11b (+) F4/80 (+) TF (+) cells were significantly lower in the SENP3 cKO mice than in fl/fl controls (Figure 2C,D).

We next examined TF expression on alveolar macrophages, which located in the alveolar space by immunofluorescence. At baseline, we could hardly observe TF staining in lung tissue, including F4/80 (+) alveolar macrophages, in either SENP3^fl/fl^ or cKO animals. Following LPS injection, lung TF expression increased in both SENP3^fl/fl^ and cKO animals, with the signal mostly located in the lung interstitium (Figure 2E). Then, we observed TF expression in F4/80 (+) alveolar macrophages and found that TF expression in these cells markedly increased in SENP3^fl/fl^ mice following LPS injection. Interestingly, TF expression in the alveolar macrophages from SENP3 cKO mice was very low (Figure 2E). Quantification of TF fluorescence intensity on F4/80‐positive alveolar macrophages after LPS injection showed that TF expression was significantly higher in SENP3^fl/fl^ than in SENP3 cKO mice (Figure 2F). These results thus suggest that SENP3 contributed to monocytes/macrophages TF production and coagulation activation in the context of LPS‐induced sepsis.

### 3.3 ROS‐induced SENP3 accumulation contributed to LPS‐induced TF production in monocytes/macrophages through enhancing JNK phosphorylation

**FIGURE 3:**
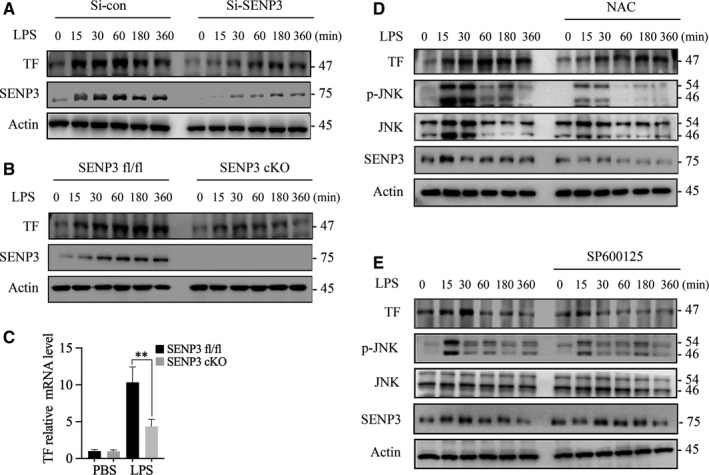
Reactive oxygen species (ROS)‐induced SENP3 accumulation contributed to lipopolysaccharide (LPS)‐induced tissue factor (TF) production in monocytes/macrophages through enhancing JNK phosphorylation. A, RAW 264.7 cells transfected using a control or SENP3‐specific siRNA were treated using LPS (1 µg/mL), after which immunoblotting (IB) was used to measure TF and SENP3 levels at the indicated time‐points. B, BMDMs from SENP3^fl/fl^ and SENP3 conditional knockout (cKO) mice were treated using LPS (1 µg/mL), after which IB was used to measure TF and SENP3 levels at the indicated time‐points. C, BMDMs isolated from SENP3^fl/fl^ and SENP3 cKO mice were treated for 4 h using LPS (1 µg/mL), after which TF expression was measured via qRT‐PCR. D, WT murine BMDMs were treated using LPS (1 µg/mL) with or without 5 mmol/L NAC (4 h pre‐treatment); SENP3, JNK, p‐JNK and TF levels were measured via IB at the indicated time‐points. E, Effects of JNK inhibitor SP600125 on RAW264.7 cell TF levels following LPS treatment. Cells were treated with LPS ± 10 µmol/L SP600125, after which IB was used to assess p‐JNK and TF at the indicated time‐points. Data are means ± SD and (A‐F) are representative of three independent experiments. *P < .05; **P < .01

To explore whether SENP3 accumulation contributed to LPS‐induced TF production, we sought to confirm a direct role for SENP3 as a mediator of LPS‐induced TF expression. The results showed that levels of TF rose in 15 minutes following LPS stimulation and lasted for 6 hours, along with SENP3 accumulation (Figure 3A,B). When SENP3 was knocked down via siRNA in RAW macrophages, LPS‐induced TF expression was significantly reduced (Figure 3A), consistent with findings in BMDMs from SENP3 cKO mice (Figure 3B). We further investigated the correlation between SENP3 and TF expression levels in SENP3^fl/fl^ and cKO murine BMDMs by quantitative RT‐PCR (qRT‐PCR). The results revealed that TF transcription was significantly decreased in the BMDMs of cKO mice compared to fl/fl control‐derived cells (Figure 3C). These results thus indicated that SENP3 promotes TF production upon LPS stimulation via inducing TF transcription in monocytes and macrophages.

It is known that the rapid accumulation of SENP3 protein level results from a blockage of degradation due to cysteine oxidation, which can be blocked by antioxidant N‐acetyl‐L‐cysteine (NAC).^13^ Lao's study proved that SENP3 selectively contributed to JNK phosphorylation, whereas ERK and p38 phosphorylation were unaffected in LPS‐activated macrophages.^15^ To test whether the blockage of ROS can abolish LPS‐induced JNK phosphorylation and TF production in macrophages, we treated BMDMs with NAC prior to LPS (1 μg/mL) treatment.^15^ We showed that NAC pre‐treatment inhibited LPS‐induced SENP3 accumulation, along with impaired JNK phosphorylation and decreased TF production in WT BMDMs (Figure 3D). The results showed that ROS and ROS‐induced SENP3 accumulation contributed to LPS‐induced TF production and JNK phosphorylation.

**FIGURE 4:**
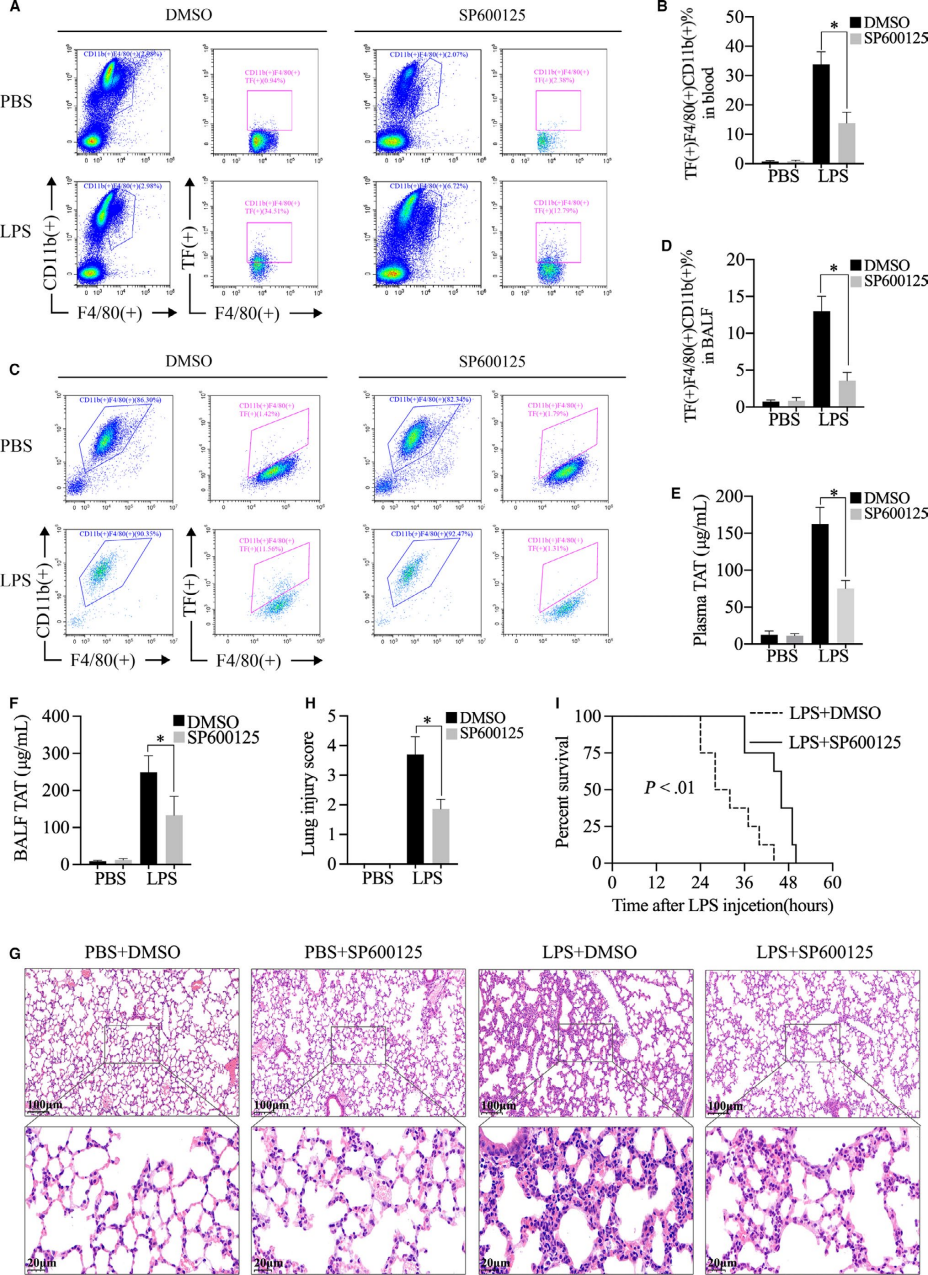
JNK inhibitor SP600125 treatment down‐regulated lipopolysaccharide (LPS)‐induced monocytes/macrophages tissue factor (TF) production and attenuated LPS‐activated coagulation. SP600125 treatment protected against LPS‐induced acute lung injury and sepsis. A‐F, Male WT C57BL/6 mice (n = 3) were injected i.p. using LPS (30 mg/kg); then, these mice were injected i.p. with SP600125 (75 mg/kg) or DMSO. A, TF in CD11b (+) F4/80 (+) peripheral blood cells at 4 h after LPS injection was assessed by flow cytometry. B, The statistics of the frequency of TF (+) CD11b (+) F4/80 (+) cells. C, TF in CD11b (+) F4/80 (+) cells from bronchoalveolar lavage fluid (BALF) at 4 h after LPS injection was assessed by flow cytometry. D, The statistics of the frequency of TF (+) CD11b (+) F4/80 (+) cells. E, The levels of TAT complexes in the plasma at 4 h after LPS injection were assessed by ELISA. F, The levels of TAT complexes in the BALF at 4 h after LPS injection were assessed by ELISA. G, H&E staining of lung tissues of the four groups at 8 h after LPS injection was accessed. H, The total lung injury score was accessed. I, Male C57BL/6 mice (n = 8 per group) were injected i.p. with LPS following SP600125 or DMSO injection and then monitored for survival every 4 h for up to 60 h. Data are means ± SD and (A‐F) are representative of three independent experiments. *P < .05

To definitively demonstrate that JNK phosphorylation downstream of TLR4 mediates TF production, we treated RAW macrophages using the specific JNK inhibitor SP600125. Following LPS treatment, cells treated with SP600125 produced significantly less TF than untreated cells (Figure 4E). Therefore, these findings demonstrated that SENP3 mediates TF expression through a JNK activation‐dependent mechanism upon LPS stimulation.

### 3.4 JNK inhibitor SP600125 treatment down‐regulated LPS‐induced monocytes/macrophages TF production and attenuated LPS‐activated coagulation: SP600125 treatment protected against LPS‐induced acute lung injury and sepsis

The above results showed SENP3 mediated TF expression through a JNK activation‐dependent mechanism upon LPS stimulation in vitro. To verify the effect of JNK phosphorylation on TF production in vivo, mice were treated with SP600125 (75 mg/kg) or DMSO after LPS injection. TF production in CD11b (+) F4/80 (+) cells from blood and BALF was accessed by flow cytometry at 4 hours after LPS injection. After SP600125 treatment, the proportion of CD11b (+) F4/80 (+) TF (+) cells in the blood (Figure 4A,B) and in the BALF (Figure 4C,D) was significantly lower than that of DMSO controls. Consistently, plasma TAT and BALF TAT were markedly decreased by SP600125 treatment (Figure 4E,F). H&E staining of lung sections revealed that the ALI pathological changes in the lung tissues of the mice injected with SP600125 were markedly attenuated (Figure 4G,H). Finally, mice injected with SP600125 showed higher survival rates than mice injected with DMSO (Figure 4I). Collectively, these data demonstrated that SP600125 treatment inhibited monocytes/macrophages TF production and attenuated LPS‐induced coagulation activation. Moreover, SP600125 treatment protected against LPS‐induced ALI and sepsis.

## 4 DISCUSSION

Together, our results suggest that LPS induces ROS‐dependent SENP3 accumulation within macrophages and monocytes. SENP3 then promotes JNK activation, leading to TF expression in these cells, thereby initiating the coagulation cascade and leading to sepsis‐induced ALI. These results highlight the importance of myeloid cell‐specific SENP3 expression as a key pathological mechanism governing sepsis‐associated coagulation activation and lung injury. Importantly, this work reveals a link between SUMOylation and coagulation during sepsis.

Reactive oxygen species‐mediated SENP3 up‐regulation and subsequent JNK activation are essential to allow ROS to modulate cellular TF expression and coagulation responses. TF has been recognized as a redox‐sensitive gene and a key link between ROS and coagulation.^19^, ^20^ The TF gene is known to have an LPS response element in its promoter region, containing binding sites for both activator protein‐1 (AP‐1) (two sites) and NF‐κB (one site); binding to both the distal AP‐1 region and the NF‐κB region is essential for maximal TF transcription.^21^ Lao et al^15^ found that transcriptional activity of AP‐1 was inhibited by SENP3 knockdown in RAW cells, whereas that of NF‐κB was not affected. Combined with these data, we may conclude that SENP3 regulates TF expression by promoting JNK activation and influences the transcriptional activity of AP‐1 in macrophages. These findings thus provide new insights into the mechanism underlying ROS regulation of TF expression and LPS‐activated coagulation.

Tissue factor expression by both haematopoietic and non‐haematopoietic cells was thought to play a significant role in the coagulation activation during endotoxemia and sepsis.^7^ However, recent studies demonstrate that TF expression by myeloid cells, especially monocytic cells, but not endothelial cells, contributes to pathological coagulation during sepsis.^5^, ^6^ Our results suggest that lack of SENP3 expression in myeloid cells can similarly reduce monocyte/macrophage TF and pathological coagulation in the same model system. This work further confirms the central role of macrophage‐ and monocyte‐derived TF in the initiation of coagulation during sepsis.

Valid therapies for coagulation abnormalities in sepsis and septic ALI are missing. Previous methods inhibiting coagulation activation all failed.^2^, ^3^ In this study, we found that selectively inhibiting JNK phosphorylation reduced TF expression and alleviated coagulation activation. These results suggest that JNK inhibition may represent a promising therapeutic strategy to alleviate septic coagulation activation.

In summary, this manuscript highlights a novel role for the SUMO protease SENP3 as a regulator of LPS‐induced monocyte and macrophage coagulation response and reveals a link between SUMOylation and coagulation.

## CONFLICT OF INTEREST

All authors including X. Chen, Y. Lao, J. Yi, J. Yang, S. He and Y. Chen confirm that there are no conflicts of interest.

## AUTHOR CONTRIBUTIONS

Y. Chen, S. He, J. Yang and J. Yi conceived and designed the experiments. X. Chen conducted the experiments, collected data and analysed data. Y. Lao provided SENP3 cKO mice. Y. Chen, S. He, J. Yang and J. Yi interpreted data and wrote the manuscript. Y. Chen and J. Yi were involved in project planning and supervision. All authors reviewed the manuscript.

## Data Availability

The data that support the findings of this study are available from the corresponding author upon reasonable request.
